# Beyond Natriuretic Peptides: Unveiling the Power of Emerging Biomarkers in Heart Failure

**DOI:** 10.3390/biom14030309

**Published:** 2024-03-06

**Authors:** Roberto Licordari, Michele Correale, Salvatore Bonanno, Matteo Beltrami, Michele Ciccarelli, Antonio Micari, Alberto Palazzuoli, Giuseppe Dattilo

**Affiliations:** 1 Department of Biomedical and Dental Sciences and Morphofunctional Imaging, Section of Cardiology, University of Messina, 98122 Messina, Italy; robertolicordari@gmail.com (R.L.); salvatore.bonanno@studenti.unime.it (S.B.); amicari@unime.it (A.M.); gdattilo@unime.it (G.D.); 2 Cardiothoracic Department, Policlinico Riuniti University Hospital, 71100 Foggia, Italy; michele.correale@libero.it; 3 Cardiomyopathy Unit, Careggi University Hospital, 50124 Florence, Italy; beltrami.matteo1@gmail.com; 4 Arrhythmia and Electrophysiology Unit, Careggi University Hospital, 50124 Florence, Italy; 5 Cardiology Division, Department of Medicine, Surgery and Dentistry, University of Salerno, 84081 Fisciano, Italy; mciccarelli@unisa.it; 6 Cardiovascular Diseases Unit, Cardio Thoracic and Vascular Department, Le Scotte Hospital, University of Siena, 84081 Salerno, Italy

**Keywords:** heart failure, cardiac biomarkers, precision medicine, non-natriuretic biomarkers, omics in heart failure

## Abstract

Heart failure (HF) represents a significant global health challenge, characterized by high morbidity and mortality rates, and imposes considerable burdens on healthcare systems and patient quality of life. Traditional management strategies, primarily relying on clinical assessments and standard biomarkers like natriuretic peptides, face limitations due to the heterogeneity of HF. This review aims to delve into the evolving landscape of non-natriuretic biomarkers and the transformative potential of omics technologies, underscoring their roles in advancing HF treatment towards precision medicine. By offering novel insights into the biological underpinnings of HF, including inflammation, myocardial stress, fibrosis, and metabolic disturbances, these advancements facilitate more accurate patient phenotyping and individualized treatment strategies. The integration of non-natriuretic biomarkers and omics technologies heralds a pivotal shift in HF management, enabling a move towards tailored therapeutic interventions. This approach promises to enhance clinical outcomes by improving diagnostic accuracy, risk stratification, and monitoring therapeutic responses. However, challenges such as the variability in biomarker levels, cost-effectiveness, and the standardization of biomarker testing across different healthcare settings pose hurdles to their widespread adoption. Despite these challenges, the promise of precision medicine in HF, driven by these innovative biomarkers and technologies, offers a new horizon for improving patient care and outcomes. This review advocates for the further integration of these advancements into clinical practice, highlighting the need for ongoing research to fully realize their potential in transforming the landscape of heart failure management.

## 1. Introduction

Heart failure (HF) is a global health concern characterized by high morbidity and mortality rates, presenting significant societal burdens in terms of healthcare costs and patient quality of life [1]. The complexity of heart failure arises from its diverse etiologies and pathophysiological mechanisms and treatment response, making its management a challenging clinical task [2].

Traditional approaches to HF management have largely relied on clinical assessment and standard biomarkers, primarily natriuretic peptides. Recognizing the heterogeneity of HF and the limitations of a one-size-fits-all treatment paradigm, there has been a shift towards precision medicine [3,4,5], which aims to tailor therapeutic strategies to individual patient characteristics, informed by a deeper understanding of disease mechanisms [6]. This approach is especially pertinent in heart failure due to its heterogeneous nature, in which patients exhibit varied responses to the common therapeutic strategy endorsed by guidelines [2,7].

This evolution has been paralleled by significant advances in molecular biology and technology, particularly the advent of omics disciplines—including genomics, proteomics, metabolomics, and transcriptomics. These fields have unveiled a plethora of non-natriuretic biomarkers that provide novel insights into the biological underpinnings of HF, encompassing aspects such as inflammation, myocardial stress, fibrosis, and metabolic disturbances [8]. The discovery and validation of these emerging biomarkers could revolutionize the management of heart failure by enabling more precise phenotyping and individualized treatment strategies [1,9].

Issues related to the standardization of biomarker measurements, the interpretation of results, and cost-effectiveness need to be addressed. Furthermore, the ethical implications of precision medicine, such as patient privacy and access to personalized therapies, are topics of ongoing debate [10].

The primary aim of this manuscript is to provide an in-depth review of the current landscape of non-natriuretic biomarkers in HF, highlighting their diagnostic and prognostic value. Additionally, it explores the impact of omics technologies on the identification of these biomarkers and discusses how these advancements are shaping the future of HF treatment within the framework of precision medicine.

## 2. Biomarkers in Heart Failure: An Overview

The emergence of biomarkers has revolutionized the diagnosis, treatment, and management of heart failure (HF), a prevalent and impactful cardiovascular condition [11].

### 2.1. Definition and Classification of Biomarkers

**Figure 1:**
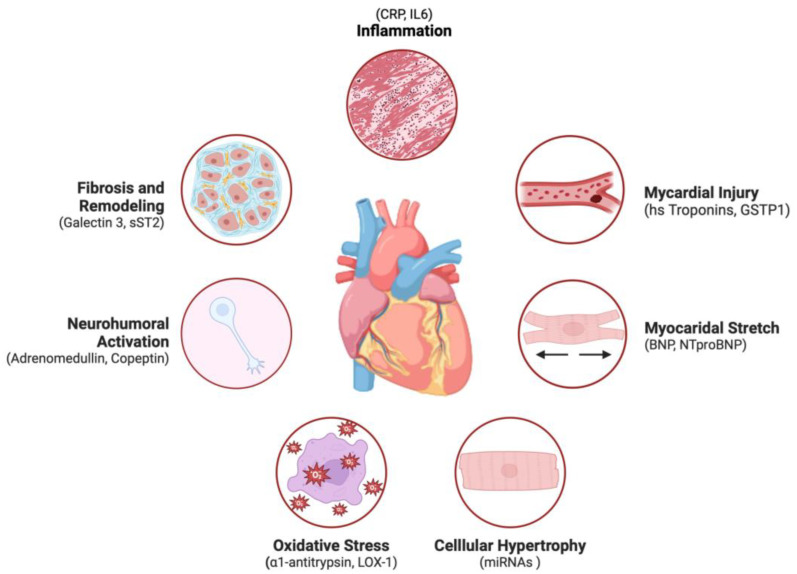
Main mechanisms involved in heart failure leading to the production of specific biomarkers.

The extensive application of biomarkers has led to a significant improvement in underlying pathophysiological mechanisms and more tailored management [12]. In HF, biomarkers are broadly classified based on the pathophysiological mechanism leading to their production and their clinical utility (Table 1, Figure 1). The main categories include natriuretic peptides (NPs) like B-type natriuretic peptide (BNP) and N-terminal pro–B-type natriuretic peptide (NT-proBNP), which are the closest to “ideal” HF biomarkers and serve as reference standards for others. These biomarkers, produced in response to cardiac stress, are crucial for diagnosing, risk stratifying, and managing HF. Another category includes neurohormonal activation markers, like norepinephrine and chromogranin, which reflect the severity of HF, and plasma renin activity (PRA), predictive of cardiac death. Adrenomedullin (ADM) and its derivative, mid-regional pro-ADM (MR-proADM), synthesized in response to volume or pressure overload, have shown promise as prognostic markers for HF [13]. Additionally, cardiac damage biomarkers, particularly cardiac troponins (TnT and TnI), are critical in the prognostic stratification of both acute and chronic HF. Moreover, markers of myocardial remodeling, inflammation, and oxidative stress emerged in the last decade [14]. This classification system underscores the multifaceted nature of heart failure and the importance of a nuanced approach to its diagnosis and management. Each biomarker category sheds light on different aspects of heart failure, contributing to a more tailored and effective treatment strategy [12].

The acknowledgment of their utility by major guidelines, such as those from the American College of Cardiology/American Heart Association (ACC/AHA) [15] and the European Society of Cardiology (ESC) [16], particularly for natriuretic peptides (NPs), marked a pivotal turn in heart failure management. These guidelines underlined the use of NPs not only for diagnosing HF but also for prognosis, guiding therapy, and even preventing left ventricular dysfunction or new-onset HF. The progression from concept to clinical utility, as proposed by the American Heart Association, emphasized the need for biomarkers to demonstrate incremental value, clinical utility, and cost-effectiveness. The evolution of biomarkers in HF reflects an intricate tapestry of pathophysiological insights, from cardiac remodeling and neurohormonal activation to myocardial injury. The current perspective on HF biomarkers is not only focused on diagnosis and management but also encompasses prevention and disease progression by a multi-marker approach, enhancing the accuracy of diagnosis and risk stratification [17].

The figure illustrates the key pathophysiological processes in heart failure that lead to the generation of specific biomarkers, including key markers for each pathophysiological mechanism.

### 2.2. Neurohormonal Activation Biomarkers

#### 2.2.1. Norepinephrine

Studies by Cohn et al. and Francis et al. have established plasma norepinephrine as a critical biomarker in chronic congestive heart failure, correlating with survival rates and the effectiveness of therapy in heart failure patients [18,19]. These findings highlight the prognostic value of norepinephrine levels in understanding the progression and management of chronic heart failure.

#### 2.2.2. Chromogranin A and B

Ceconi et al. and Røsjø et al. identified Chromogranin A as a novel neurohumoral factor in heart failure and a predictor of mortality [20,21]. Chromogranin B was recognized for its expression in the failing myocardium, suggesting its potential as a cardiac biomarker. Chromogranin A emerged as a good predictor of mortality in patients with chronic HF (Table 2).

#### 2.2.3. Plasma Renin Activity (PRA)

In the case of PRA, the supporting evidence is not homogeneous. In a study, plasma renin activity (PRA) predicted cardiac death independently of NT-proBNP and LVEF [22]. In the Aliskiren Trial on Acute Heart Failure Outcomes (ASTRONAUT) study, low-baseline PRA predicted mortality and HF hospitalization. In this study, PRA reduction during therapy with aliskiren, a direct renin inhibitor, did not predict a better outcome [23].

#### 2.2.4. Adrenomedullin (ADM)

Adrenomedullin (ADM), a hormone predominantly produced in the adrenal medulla, heart, lungs, and kidneys, is secreted in response to increased volume or pressure. It exhibits various beneficial effects such as vasodilation, sodium excretion, positive inotropy, and cardioprotection [24]. Plasma levels rise in HF, yet measuring it is challenging due to its short half-life. A segment of its precursor, the midregional pro-adrenomedullin (MR-proADM), is simpler to measure and has been evaluated as a predictive indicator for HF [24]. The BACH trial identified MR-proADM as a better predictor of 90-day mortality compared to BNP in acute HF [25]. This was further validated by a secondary analysis in the PRIDE study, which found MR-proADM to be the most accurate predictor of mortality within a year of acute HF diagnosis. In contrast, MR-proANP and NT-proBNP were more effective in prognostication beyond the first year post-diagnosis [26]. Although these findings endorse MR-proADM’s role as a prognostic marker, especially for short-term risk assessment, its non-cardiac specificity limits its clinical application.

#### 2.2.5. Copeptin

Vasopressin is a hormone produced and released by the hypothalamus with antidiuretic and vasoconstrictive activities in response to hyperosmolarity or hypovolemia [27]. In HF, levels of vasopressin rise due to the stimulation of baroreceptors, which is a response to decreased cardiac output mimicking a state of hypovolemia [27]. Copeptin, the C-terminal segment of pro-vasopressin, can be measured more easily than vasopressin itself [28]. The BACH study revealed that patients with higher copeptin levels experienced more severe lung and limb fluid accumulation and had a higher mortality rate within three months [28]. Furthermore, a meta-analysis involving patients with both acute and chronic HF indicated that copeptin is as effective as NTproBNP in predicting overall mortality [29].

#### 2.2.6. Endothelin-1 (ET-1)

Endothelin-1 (ET-1), a substance produced by the vascular endothelium, is generated in response to factors such as shear stress and inflammation. ET-1 is known for its roles in vasoconstriction, promoting inflammation, oxidative actions, and contributing to cardiac remodeling. This compound is initially released as a precursor known as big ET-1, from which the C-terminal fragment is subsequently cleaved [30]. In a detailed analysis from the Acute Study of Clinical Effectiveness of Nesiritide in Decompensated Heart Failure (ASCEND-HF), it was observed that the baseline levels of ET-1 were linked to increased in-hospital adverse events and mortality at three months in patients admitted with acute HF, adding to the prognostic value provided by NT-proBNP [31]. Notably, this includes not just the initial levels, but also the decline over time in ET-1 that appears to be indicative of more favorable outcomes [32].

#### 2.2.7. Urocortin-1

Urocortin-1 belongs to the corticotropin-releasing factor family and is primarily synthesized in the central nervous system. Vasodilation, inotropic, and cardioprotective effects are the main effects of urocortin-1 [33]. In the context of HF, levels of plasma urocortin-1 are found to increase. However, when it comes to its role as a biomarker, urocortin-1 does not seem to offer extra diagnostic or prognostic utility beyond what NT-proBNP already provides [33].

### 2.3. Biomarkers of Cardiac Damage

Elevated levels of myocardial injury biomarkers have been observed in patients with heart failure (HF). These elevations may arise from tissue ischemia, a consequence of both coronary and non-coronary artery diseases, or from cell death caused by factors such as neurohormonal activation, inflammation, or apoptosis [34]. Regardless of the underlying cause, this increase signifies a range of pathophysiological processes, including the destabilization of membrane lipid layers due to lipid peroxidation and cellular destruction via necrosis or apoptosis [35]. While the disruption of the normal cardiac myocyte membrane leads to the release of various cellular and structural proteins like cardiac fatty acid binding protein, creatine kinase, myoglobin, and troponins, it is primarily the latter that has become the benchmark for diagnosing myocardial infarction [36].

#### 2.3.1. Cardiac Troponins

Cardiac troponins, specifically troponin I (TnI) and troponin T (TnT), are crucial biomarkers used in the clinical assessment of the heart, particularly in acute coronary syndromes and HF [37]. As components of the cardiac muscle contractile apparatus, their presence in the blood is indicative of myocardial injury or necrosis, but the chronic release of cytoplasmic vesicles (blebs) containing cellular material has also been demonstrated. The recent advancements in high-sensitivity assays for detecting these biomarkers have greatly enhanced their diagnostic and prognostic utility in heart failure management.

Elevated levels of cardiac troponins, especially when measured through high sensitivity assays, have been shown to be highly predictive of both systolic and diastolic cardiac dysfunction, and are closely associated with conditions that predispose to heart failure, like aortic stenosis and stable coronary artery disease. In the realm of heart failure, several studies have shed light on the significance of these biomarkers.

For instance, Latini et al. found that using standard assays for cardiac troponins, only 10% of chronic HF patients had detectable levels of TnT, and these were linked to increased risks of death and hospital readmissions. However, when high-sensitivity cardiac troponin assays were employed, nearly 92% of the same cohort showed detectable levels of TnT, underscoring the enhanced sensitivity and predictive power of these advanced assays, and TnT was predictive of all-cause death [38].

Further emphasizing their role in prognosis, clinical studies have demonstrated that patients with heart failure exhibiting elevated baseline concentrations of high-sensitivity cardiac troponins (hs-cTn) are at a higher risk of cardiovascular mortality and poor cardiovascular outcomes. For example, heart failure with reduced ejection fraction (HFrEF) patients showing the most elevated hs-cTnT concentrations had significantly increased risks of cardiovascular death and heart failure hospitalization. Moreover, a comprehensive approach, including hs-cTnT and hs-cTnI alongside NT-proBNP and clinical features, significantly improved the risk prediction of outcomes in patients with both acute and chronic heart failure [39,40].

Furthermore, research has shown that elevated cardiac troponins in acute and chronic heart failure patients are of prognostic significance. In a study of 144 patients with acute heart failure, more than 99% had hs-TnI levels above the 99th percentile of the reference population, with levels > 23 ng/L being associated with an increased risk of hospitalization or death [41]. Similar results were described by Pascual-Figal et al. [42].

The use of cardiac troponin to guide HF therapy is a topic under debate. The therapeutic strategy of HFrEF management was found to significantly reduce the concentrations of hs-cTnT improving survival, but SGLT2 inhibitors exerted favorable effects on HFrEF/HFpEF and renal outcomes, independent of baseline hs-cTnT concentrations [43,44].

Guidelines for the approach for cardiac troponins usage in HF patients are different between ACC/AHA and ESC. ACC/AHA guidelines recommend troponin levels at admission in all patients with HF (class I, LOE A) for the purpose of risk stratification [15]. Conversely, ESC guidelines recommend its use only at admission in patients with the suspicion of acute HF within a complete laboratory analysis panel (class I, LOE C), with the main goal of excluding an acute coronary syndrome [16].

In summary, cardiac troponins, particularly with the advent of high-sensitivity assays, have emerged as important biomarkers in the management of heart failure. Their ability in stratifying risk in patients with both acute and chronic heart failure underscores their importance in both the diagnosis and prognosis of heart failure.

#### 2.3.2. Heart-Type Fatty-Acid-Binding Protein (H-FABP)

H-FABP, primarily found in the heart, is involved in the transport of fatty acids, a critical function in myocardial energy metabolism. Molecularly, H-FABP is a small, cytosolic protein that responds swiftly to myocardial damage, thus serving as an early marker of cardiac injury. Its role in heart failure is underscored by its rapid release into circulation following an ischemic insult, preceding the elevation of troponins [35,45].

From a clinical perspective, H-FABP has been the subject of numerous studies. For instance, research has shown its efficacy in the early diagnosis of acute myocardial infarction, often manifesting elevated levels before troponin. Furthermore, it holds potential in risk stratification, particularly in acute coronary syndromes (ACS), where its levels have been correlated with adverse outcomes [46,47]. The diagnostic and prognostic value of H-FABP in HF, especially when used in conjunction with other biomarkers like troponins, has been increasingly recognized in recent works in the literature [48,49].

#### 2.3.3. Glutathione Transferase P1 (GSTP1)

GSTP1, an enzyme involved in detoxification processes, has gained attention as a biomarker for oxidative stress and myocardial damage. Its role is particularly intriguing in the context of heart failure, where oxidative stress plays a significant pathophysiological role. GSTP1’s expression in cardiac tissues reflects the body’s response to oxidative injury and myocardial stress.

Clinically, elevated levels of GSTP1 have been associated with various aspects of heart failure. Studies have explored its correlation with the severity of HF, indicating its potential as a prognostic marker [50]. The relationship between GSTP1 levels and cardiac function has also been a subject of research, suggesting that this biomarker could offer valuable insights into the severity and progression of HF [51]. The integration of GSTP1 in the biomarker panel for HF could thus provide a more comprehensive understanding of the disease process and aid in tailoring more effective treatment strategies.

### 2.4. Markers of Myocardial Fibrosis

#### 2.4.1. Galectin-3

Galectin-3, a β-galactoside-binding lectin, plays a pivotal role in controlling myocardial and microvascular inflammation. It influences the migration of mononuclear cells and promotes the excessive production of collagen and fibroblast proliferation. These activities contribute to adverse cardiac remodeling and dysfunction [52]. The secretion of galectin-3 by macrophages is thought to be primarily triggered by aldosterone. This hormone mediates the effects of autocrine and paracrine signals from transforming growth factor-β and cyclin D1 in fibroblasts, which in turn moderates the proliferation of myofibroblasts, the recruitment of immune cells, and the deposition of the extracellular collagen matrix [53]. The secretion of Gal-3, a biomarker of heart failure, is influenced by various factors, including aldosterone, Angiotensin II, hypertension, and myocardial injury, underlining its multifaceted role in heart failure pathophysiology. Additionally, the expression of galectin-3 mRNA in the myocardium and vascular system has been linked to the influence of pro-inflammatory cytokines, such as interferon-gamma and interleukin-6 [54].

While galectin-3 is found in higher levels in HF, it is not a reliable biomarker for diagnosing acute HF. However, it has been identified as a significant predictor of re-hospitalization after discharge in patients with acute HF [55]. The GALectin-3 in Acute heart failure (GALA) study revealed that levels of galectin-3 measured upon admission were effective in predicting mortality within 30 days, but not over a 1-year period [56]. Conversely, sub-analyses of the RELAXin in Acute Heart Failure (RELAX-AHF) and the ProBNP Outpatient Tailored Chronic Heart Failure (PROTECT) did not demonstrate its predictive value for mortality at the 6-month mark [57].

While galectin-3 is a significant biomarker, its prognostic capability in HFrEF and HFpEF is not as robust as NT-proBNP or sST2, possibly due to its broader biological roles. The prognostic accuracy of Gal-3 in heart failure is influenced by multiple factors, such as renal function (i.e., chronic kidney disease or hemodialysis) and respiratory impairment (COPD or Pneumonia), which highlights the need for a multifactorial approach in evaluating its prognostic value [57]. Despite this, the ACC/AHA guidelines do recommend galectin-3 measurement for prognostic stratification in chronic HF patients [15]. However, the European Society of Cardiology (ESC) guidelines refrain from recommending its use in clinical practice due to a lack of robust evidence [16].

#### 2.4.2. Soluble Isoform of Suppression of Tumorigenicity 2

The Suppression of Tumorigenesis-2 ligand (ST2L), part of the Toll-like receptor family, interacts with interleukin 33 (IL-33). The IL-33/ST2L pathway primarily functions within the immune system but also exerts anti-apoptotic, anti-fibrotic, and anti-hypertrophic effects on the heart. sST2, serving as a decoy receptor for IL-33, is primarily produced outside the heart in reaction to hemodynamic stress, inflammation, and pro-fibrotic stimuli. Overall, sST2 showcases the key attributes of an ideal biomarker: it provides high accuracy in a single measurement, can be measured repeatedly for risk stratification when used in conjunction with multiple biomarkers, and is readily available in clinical practice at a feasible cost [alm-43-3-225.pdf 68] [58].

As sST2 is not specific to cardiac conditions, it cannot be employed for the diagnosis of HF. However, it is valuable in risk stratification. Levels of sST2 at both admission and discharge have been shown to predict all-cause and cardiovascular mortality in acute HF patients, as confirmed by a meta-analysis [59]. The importance of monitoring sST2 levels during HF hospitalization was underscored in a study involving 150 acute HF patients, where the percentage change in sST2 predicted 3-month mortality, irrespective of BNP or NT-proBNP levels [60]. The Translational Initiative on Unique and novel strategies for the Management of Patients with Heart failure (TRIUMPH) cohort study, involving 496 acute HF patients with seven assessments over a year, found similar results. Baseline sST2 levels predicted all-cause death or HF hospitalization, and changes in sST2 levels over time were even more predictive, independent of NT-proBNP measurements [61].

sST2 is crucial for risk stratification in chronic HF patients. Its prognostic value in chronic HF is independent of NT-proBNP and hs-TnT levels and is less affected by age compared to these biomarkers [62]. sST2 outperforms galectin-3 in this respect. Furthermore, sST2 independently predicts reverse remodeling and is included in the ST2-R2 score, which comprises several factors including sST2 levels below 48 ng/mL [58].

There is no consensus on the optimal prognostic cutoff for sST2 in chronic HF. Elevated sST2 levels, specifically above 35 ng/mL, have been recognized as effective and specific markers for predicting all-cause mortality, cardiovascular death, and hospitalization [62,63], so this has become the most frequently used cutoff.

Similar to Galectin-3, the ACC/AHA guidelines recommend measuring sST2 for the prognostic evaluation of chronic HF patients [15], while the ESC guidelines do not endorse its use due to insufficient evidence [16].

#### 2.4.3. Growth Differentiation Factor-15

Growth Differentiation Factor-15 (GDF15), part of the transforming growth factor-beta superfamily, plays a key role in regulating inflammation and tissue repair [64]. GDF-15 targets several molecular pathways, including the inhibition of c-Jun N-terminal kinase, Bcl-2-associated death promoter, and epidermal growth factor receptor, while activating Smad/eNOS. The PI3K/AKT signaling pathways are also pivotal in its function, providing protection to both progenitor and mature endothelial cells. GDF15’s cardioprotective influence is composed of anti-inflammatory, antioxidative, and antiapoptotic effects [65].

In cardiomyocytes, GDF15 expression significantly increases in response to ischemia and reperfusion injury and is associated with cardiac fibrosis following inflammation caused by acute myocardial infarction (AMI) and heart failure (HF).

While GDF-15 is not exclusive to cardiac tissue, its increased plasma levels have been shown to play a prognostic role in HF [66]. For instance, in a sub-analysis of the RELAX-AHF study, an elevation in GDF-15 levels during HF hospitalization (rather than the levels at admission) was predictive of cardiovascular death at 180 days [67]. In a separate study involving 455 chronic HF patients, GDF-15 was found to predict mortality, independent of other clinical and laboratory factors, including NT-proBNP [68]. Additionally, a post hoc analysis of the Val-HeFT trial demonstrated that changes in GDF-15 levels over a year remained an independent predictor of mortality [69].

#### 2.4.4. Wnt-β Catenin

The Wnt-β catenin pathway is integral to various cellular processes, including embryonic development, cell proliferation, and differentiation [70]. At the molecular level, it involves a complex network of proteins that regulate gene transcription, cellular growth, and tissue remodeling. These effects are mediated by three different pathways.

Canonical Wnt/β-Catenin Pathway: This pathway is activated by the binding of Wnt to Frizzled receptors and LRP5/6 co-receptors, leading to the stabilization and nuclear translocation of β-catenin. In the nucleus, β-catenin associates with TCF/LEF transcription factors to regulate gene expression. This is the most studied and well-known pathway.

Non-Canonical Planar Cell Polarity Pathway: This pathway operates independently of β-catenin and involves the activation of small GTPases and JNK, influencing cell movement and polarity.

Non-Canonical Wnt/Ca^2+ Pathway: Triggered by Wnt-Frizzled interactions, this pathway leads to increased intracellular calcium levels and activates calcium-dependent signaling cascades, impacting various cellular functions.

In the context of cardiac fibrosis, the Wnt-β catenin pathway interacts with several other signaling pathways, notably adenosine and TGF-β, to mediate fibrotic responses [70]. Adenosine, a purine nucleoside, plays a crucial role in cardiac physiology and pathology. It functions through four G-protein-coupled adenosine receptors (A1, A2A, A2B, and A3), influencing processes like myocardial oxygen consumption, coronary blood flow, and cardiomyocyte function [71]. In cardiac fibrosis, adenosine signaling has been shown to modulate fibroblast activity and collagen deposition, both key aspects of the fibrotic process. The potential interaction between adenosine and Wnt-β catenin signaling is complex, often involving a network of signaling cascades that converge on common molecular targets such as PI3K/Akt, influencing cell survival, proliferation, and fibrosis [71,72].

The TGF-β pathway is a major regulator of fibrosis, influencing the differentiation of fibroblasts into myofibroblasts, cells that are central to the fibrotic process. TGF-β stimulates the synthesis of extracellular matrix proteins and inhibits their degradation, thereby contributing to the pathological accumulation of fibrous tissue in the heart. The cross-talk between TGF-β and Wnt-β catenin pathways is pivotal in cardiac fibrosis. TGF-β can activate the canonical Wnt pathway, leading to β-catenin accumulation and activation. This cross-regulation amplifies the fibrotic response, contributing to the excessive deposition of the extracellular matrix in the myocardium [73].

The expression of β-catenin could potentially be a useful biomarker for identifying patients who may benefit from anti-Wnt/β-catenin therapy. However, further studies and investigations are needed to fully understand the regulatory mechanism of the Wnt/β-catenin signaling pathway and its role in cardiac disease progression, including its potential as a heart failure biomarker.

#### 2.4.5. Non-Coding RNAs

Non-coding RNAs (ncRNAs), particularly microRNAs (miRNAs) and long non-coding RNAs (lncRNAs), continue to be pivotal in regulating cardiac gene expression, impacting disease progression and therapeutic outcomes in heart failure (HF) [74]. miRNAs such as miR-208a, miR-208b, and miR-499 remain crucial for cardiac hypertrophy and remodeling, influencing post-transcriptional gene regulation [75,76]. Recent research has further highlighted the significance of lncRNAs, including ZFAS1, H19, and Miat, in cardiac pathology. ZFAS1 has been associated with intracellular Ca^2+^ overload and impaired contractile function [77], while H19’s role in cardiac remodeling and fibrosis, and Miat’s involvement in myocardial infarction risk [78], underscore their potential as therapeutic targets and diagnostic biomarkers.

Emerging evidence also points to the therapeutic potential of targeting specific ncRNAs in HF. This indicates a promising avenue for miRNA-based therapeutic strategies [79]. Furthermore, the identification of circulating ncRNAs as biomarkers opens new pathways for early HF diagnosis, enhancing the molecular understanding of the disease and paving the way for personalized medicine.

This evolving understanding, enriched with recent advancements, underscores the need for continued research into ncRNAs’ roles, aiming to harness their full potential for diagnostic, prognostic, and therapeutic applications in HF.

### 2.5. Biomarkers of Inflammation

Inflammation in heart failure (HF) can originate from direct damage to cardiomyocytes, such as that caused by ischemia or pressure overload, or it might reflect a systemic inflammatory condition associated with comorbidities [13]. Traditional inflammatory biomarkers, including tumor necrosis factor-alpha and its soluble receptor I, interleukin-6, metalloprotease 17 (ADAM-17), and cluster of differentiation 146 (CD146), have not demonstrated independent predictive value beyond clinical observations, echocardiographic data, and natriuretic peptides (NPs). Furthermore, these markers are cleared by the kidneys, which is considered a disadvantage for patients with HF. As a result, their diagnostic effectiveness is considerably lower than that of BNP and NT-proBNP [80].

The initial evidence of C-reactive protein (CRP) elevation in heart failure (HF) can be traced back more than 60 years [81]. Subsequent research has extensively explored this relationship, underscoring the prognostic significance of CRP, tumor necrosis factor alpha (TNFα), and interleukin-6 (IL-6) in chronic HF [82]. The increase in these three biomarkers has also been linked to a heightened risk of developing HF [83,84]. More recently, cancer antigen 125 (CA-125) has gained interest. CA-125, a glycoprotein produced by mesothelial cells in response to elevated hydrostatic pressure or inflammation, is primarily known as a biomarker in cancer detection and prognosis, especially for ovarian cancer [85]. In HF, CA-125 levels have been shown to correlate with symptoms and signs of congestion. A recent multicenter study involving patients with worsening HF demonstrated its association with increased mortality and the risk of HF hospitalization after one year [85].

Although oxidative stress biomarkers were highly regarded two decades ago due to the simplicity of their measurement and the clear understanding of their role in HF pathogenesis, many, including ceruloplasmin, myeloperoxidase, and thioredoxin 1, have not shown the necessary accuracy, predictive capability, reproducibility, and reliability for clinical application. Recent oxidative stress biomarkers, like α1-antitrypsin and lectin-like oxidized low-density lipoprotein receptor-1, however, are emerging as promising components in the management of HF [86].

#### The Role of Insulin

Recent studies have underscored the significant impact of insulin resistance on heart failure, highlighting its role as an emerging biomarker. Insulin resistance, characterized by a diminished response to insulin in regulating glucose levels in the body, has been shown to affect left ventricular ejection fraction (LVEF), particularly after submaximal work [87]. This effect is notably more pronounced in insulin-resistant individuals, suggesting a direct correlation between insulin resistance and heart failure severity. The use of the euglycemic hyper-insulinemic clamp technique or, alternatively, the Homeostatic Model Assessment (HOMA) model, offers a precise quantification of insulin resistance, presenting a new avenue for heart failure assessment and management.

Moreover, the pathophysiological role of insulin resistance in heart failure is further evidenced by the efficacy of metformin. As an insulin-sensitizing agent, metformin has been demonstrated to reduce morbidity and mortality in heart failure patients [88], reinforcing the critical link between metabolic regulation and cardiovascular health. These insights advocate for a broader consideration of metabolic factors, such as insulin resistance, in the diagnosis and treatment of heart failure, promising avenues for enhancing patient care through precision medicine.

## 3. Multi-Marker Testing

HF is a complex syndrome involving diverse pathophysiological interplays, from cardiac remodeling to altered renal function and neurohormonal activation [89]. To address this complexity, a multi-marker approach, incorporating various biomarkers targeting different pathological pathways, could have a role in risk stratification and HF management.

Ky et al. examined seven biomarkers representing distinct pathophysiological pathways in HF. These included hs-CRP, BNP, myeloperoxidase, troponin I, sST2, creatinine, soluble fms-like tyrosine kinase receptor-1, and uric acid. Authors hypothesized that assessing these markers collectively could predict the risk of adverse outcomes such as mortality, cardiac transplantation, or the need for ventricular assist devices. Impressively, the combined multimarker score outperformed the Seattle HF model (SHFM), a commonly used clinical risk score in HF [90].

Further supporting this strategy, data from the RELAX-AHF trial analyzed NT-proBNP, hscTnT, sST2, GDF-15, hs-CRP, galectin-3, and cystatin-C, concluding that a serial evaluation of these markers offered a prognostic improvement when compared to a single marker strategy [91].

Currently, NPs remain the gold standard in HF prognosis, diagnosis, and management. Integrating NPs with markers of myocardial injury and fibrosis, mainly sST2 and galectin-3, is a strategy to improve risk stratification, as suggested by the ACC/AHA Guidelines [15].

Studies have investigated new biomarkers like mid-proANP, MR-proADM, pro-endothelin, and copeptin. MR-proADM, in combination with copeptin and cTnT, showed an improved discriminative value when used alongside NPs [92]. Future research combining MR-proADM with NPs, copeptin, and cardiac troponin could further enhance this approach.

Despite the potential, the multi-marker approach faces challenges, including a lack of consistent evidence in improving cardiovascular mortality and outcomes [35]. However, markers like cardiac troponins, sST2, and galectin-3 have been shown to enhance the prognostic utility of NPs in HF-associated readmission and cardiovascular mortality [66].

Machine learning and artificial intelligence, which are becoming a part of everyday life, have also emerged as key players in research within the context of a multi-marker approach in HF. For example, Chirinos et al. investigated 49 plasma biomarkers in HF patients from the Treatment of Preserved Cardiac Function Heart Failure with an Aldosterone Antagonist (TOPCAT) trial [93]. They discovered that a model comprising fibroblast growth factor-23, osteoprotegerin, tumor necrosis factor-alpha and its soluble receptor I, interleukin-6, YKL-40, fatty acid binding protein-4, GDF15, angiopoietin-2, matrix metalloproteinase-7, sST-2, and NT-proBNP effectively predicted outcomes in patients with HFpEF. Furthermore, incorporating sST2 into various studies, including the Penn HF Study, the Barcelona Study, and the ProBNP Outpatient Tailored Chronic Heart Failure (PROTECT) biomarker sub-study, enhanced the discriminative power of these models, though the optimal panel of markers is still under investigation [94].

Most emerging biomarkers are non-specific and associated with other tissues and organs, making their prognostic utility uncertain. However, subjecting these biomarkers to rigorous comparison with established markers like NPs and cTns, and statistically discriminating them, appears clinically sensible. This leads to the development of a multi-marker system, a focus of current and future research. As HF involves various processes and mechanisms, integrating newer markers with established ones could provide superior predictive value in HF management.

## 4. The Advent of Omics in HF

Most of the recognized biomarkers mentioned earlier were discovered through traditional, hypothesis-driven research. The emergence of omics, a cutting-edge approach exploring complex biochemical interactions, has provided new avenues for monitoring heart failure, including biomarker identification [95,96,97]. In HF, it is crucial to go beyond using biomarkers solely for diagnosis and prediction. The focus is now on integrating them for selecting and adjusting treatment strategy [98].

**Figure 2:**
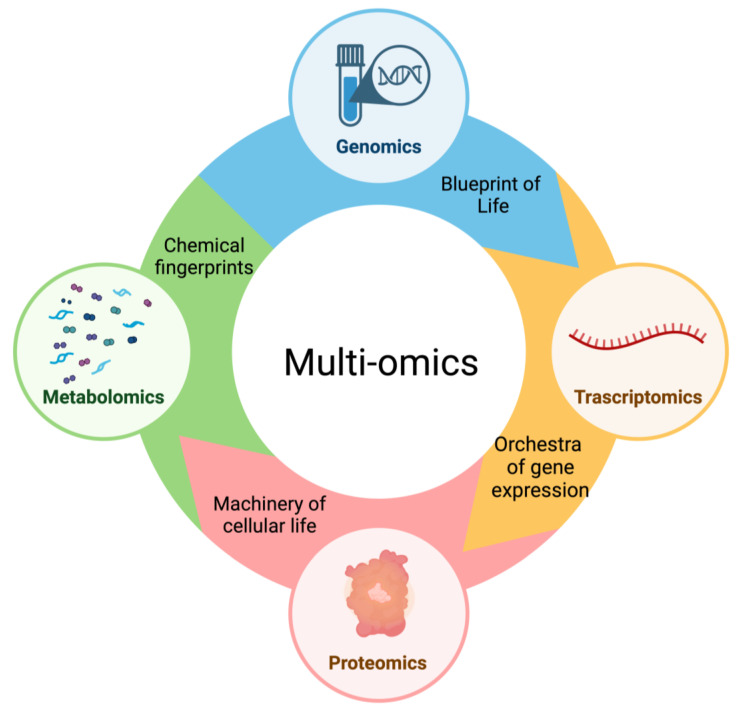
The types of omics that contribute to integrated multi-omics.

This approach involves various biological disciplines, covering the genome, transcriptome, proteome, metabolome, epigenome, and microbiome to assess a wide range of biomolecules (Figure 2) [99]. These methods originated with the Human Genome Project, leading to remarkable advancements in technologies for examining nucleic acids, proteins, and metabolic analytes at a large scale [100]. Omics could provide two key insights: firstly, therapeutic interventions can target molecules casually involved in HF pathogenesis, and secondly, modified protein molecules responding to the disease can serve as stage-specific markers [101].

This figure illustrates the interconnected landscape of ‘multi-omics’, where each sector contributes with unique insights into cardiovascular precision medicine. They each provide a comprehensive view of the molecular intricacies of health and disease.

### 4.1. Genomics

Genomics has been applied in successful research known as genome-wide association studies (GWAS) to understand the role of genes in heart failure (HF). For instance, F Dominguez described how a form of dilated cardiomyopathy (DCM), caused by mutations in BAG3, is more likely to lead to progressive heart failure in individuals over 40 years old [102]. Shah S et al. identified certain gene locations (KLHL3 and SYNPOL2–AGAP5) linked to heart failure, and also found associations between genes BAG3 and CDKN1A with left ventricular systolic dysfunction [103]. Numerous other GWAS analyses have revealed significant connections between various DNA differences and heart failure, as well as other cardiovascular diseases [104,105]. The development of new risk scores, considering genetic and epigenetic factors, looks promising in predicting the risks of heart failure and coronary heart disease [106]. In a study by Dumeny et al., genes NR3C2 and CYP11B2, related to spironolactone, were found to be associated with a better response in patients with diastolic heart failure [4].

### 4.2. Epigenomics

Epigenomic regulation plays a pivotal role in the development of HF [107]. Epigenetic mechanisms are often influenced by environmental factors, providing a pathway for the gene–environment interactions that are key contributors to the disease’s pathogenesis. These regulatory processes include various mechanisms such as DNA methylation, modifications of histones (including acetylation, methylation, and phosphorylation), chromatin remodeling, and the involvement of non-coding RNAs (encompassing microRNAs, long non-coding RNAs, and small interfering RNAs). Movassagh et al. discovered distinct histone methylation patterns linked to HF [108]. Following this, Haas J et al. successfully generated and analyzed genome-wide profiles of DNA methylation at a lower resolution in patients with dilated cardiomyopathy [109]. Meder et al. have made significant strides by comprehensively mapping DNA methylation in the human heart and identifying novel loci associated with HF [110].

### 4.3. Transcriptomics

The transcriptome provides unique insights into genetic variant functions due to its dynamic nature in responding to environmental stresses. Transcriptomics research is crucial in identifying key transcripts and genes that play a role in the pathogenesis of HF. High-throughput RNA-seq analysis has revealed alterations in genes related to the cytoskeleton and nucleocytoplasmic transport, among other critical pathways in HF [111,112,113]. Furthermore, Schiano et al. have identified specific changes in the transcriptomes of HF patients, leading to the discovery of novel genes linked to human heart tissue and the development of HF [114]. Additionally, the identification of a transcriptome biomarker panel capable of distinguishing between heart failure with reduced ejection fraction (HFrEF) and heart failure with preserved ejection fraction (HFpEF) has been achieved through gene expression microarray analysis [115]. The transcriptomic analysis of human dilated cardiomyopathy indicates a consistent and distinct gene expression pattern in HF, suggesting that a gene expression signature could potentially predict the progression of the disease.

### 4.4. Proteomics

This area of study focuses on analyzing either the entirety or a large segment of proteins in cells or tissues. Proteomes are dynamic, constantly adapting to various physiological states such as different cell cycle stages, the aging of cells, and external stressors. In heart failure (HF), specific proteomic patterns and expressions become apparent, underscoring the importance of proteomics in understanding this condition. By examining proteome changes, researchers can gain molecular-level insights into HF and its underlying causes. Despite the intricate nature of plasma proteomics, notable progress has been achieved. A prime example is the identification of quiescin Q6 sulfhydryl oxidase 1 (QSOX-1) as a crucial biomarker for detecting acute decompensated HF [116]. Furthermore, proteomic studies have revealed significant changes in proteins related to areas like the extracellular matrix, cardiomyocyte cytoskeleton, defense mechanisms, contractility, and energy metabolism, all of which exhibit altered regulation in HF [117,118]. Stenemo et al. pinpointed proteomic markers that can predict the onset of HF, independent of established risk factors [119]. Nine peptides were linked to processes such as apoptosis, inflammation, and tissue remodeling, highlighting proteomics’ potential in shedding light on HF [120].

Several studies regarding both classic and novel biomarkers in HF can be attributed to proteomic analysis. For instance, Natalia Lopez-Andrès et al. associated elevated levels of galectin-3 (Gal-3) and N-terminal propeptide III procollagen (PIIINP), along with reduced metallic metalloproteinase-1 (MMP-1), with poor long-term HF outcomes [121]. Julio Núñez et al. proposed that CA125 serves as an indicator of fluid overload, making it a valuable tool for directing decongestion therapy, as evidenced by improved eGFR in acute HF patients with renal dysfunction following a CA125-guided diuretic approach [122]. Proteomics could play a key role in refining therapeutic strategies, exemplified by Hanna K Gaggin et al.’s finding that measuring the soluble suppression of tumorigenesis (sST2) can identify chronic HF patients who might benefit from higher doses of beta-blockers [123]. The challenge, however, remains in honing the analytical methods and affirming their clinical significance.

### 4.5. Metabolomics

Metabolomics serves as a closer indicator of gene and protein functional activity, bridging the gap between genetic sequences and cellular physiology [124]. Advancements in technologies such as nuclear magnetic resonance (NMR) spectroscopy and mass spectrometry (MS) have enabled metabolomics to generate a vast array of biological markers from a single bio-sample. This has brought unprecedented precision to HF research and treatment. Metabolomics stands out for its capacity to complement other cellular processes like the extensive genetic information from human DNA sequencing, along with integrating the effects of environmental factors such as diet, physical health, microbiota changes, and toxin exposures.

While current metabolomics research in HF is still evolving, significant findings have been made. For example, Hunter et al. discovered that long-chain acylcarnitines are closely associated with HF, distinguishing between HFrEF and HFpEF patients [125]. The HF-ACTION study further revealed that elevated levels of C16 and C18:1 acylcarnitines in end-stage HF patients, as opposed to those with chronic systolic HF, correlated with a higher risk of readmission and mortality [126]. Interestingly, metabolomic profiling after left ventricular assist device implantation in end-stage HF patients showed a reduction in circulating long-chain acylcarnitines, suggesting that metabolomic profiling could be pivotal in HF management.

Metabolomics also delves into the study of small organic compounds within metabolic pathways, crucial in understanding heart failure. The analysis of enormous databases of metabolites has unveiled significant insights into the metabolic shifts occurring in HF. Patient serum and breath analyses have led to the development of metabolic profiles for systolic HF, aiding in clinical diagnosis and prognosis [127]. Du Z et al. have identified specific metabolites, including 3-hydroxybutyrate, acetone, that are elevated in different types of HF, offering predictive value for patient outcomes [128]. Wang Li et al. found distinct metabolic markers in HFrEF patients with ischemic origins [129], and Desmoulin F et al. highlighted the prognostic significance of plasma lactate to total cholesterol ratio in acute HF [130].

The multi-omics approach overcomes the limitations of single-omics analyses by providing a holistic view of the disease process. This integrative strategy can improve the diagnosis, treatment, and prognosis of HF, tailoring interventions to individual patient profiles.

## 5. Shifting to Therapeutic Targets

Recent advancements in the field of biomarkers have not only enhanced our diagnostic capabilities but also opened new avenues for therapeutic interventions. Notably, the exploration of natriuretic peptides, traditionally regarded as key biomarkers in heart failure management, has extended into their potential as therapeutic agents. An innovative example of this approach is the extraction of natriuretic peptides from natural sources, such as snake venom, illustrating their dual role in both signaling and therapeutic domains [131]. This approach underscores the possibility of repurposing biomarkers, traditionally used for diagnosis, as novel therapeutic options.

By leveraging omics technologies, Kolur et al., performing a bioinformatics analysis on the GSE141910 dataset from the Gene Expression Omnibus (GEO), also identified therapeutic targets, underscoring the role of certain key genes and pathways in HF’s progression [132]. This dual functionality underscores a paradigm shift in heart failure management, where biomarkers emerge as central figures both in understanding the disease and in crafting tailored therapeutic strategies.

## 6. Conclusions

The landscape of HF management has been significantly enriched by the discovery and implementation of biomarkers. Despite their proven utility in diagnosing and prognosticating HF, the integration of novel biomarkers into routine clinical practice encounters several challenges. The foremost among these is the variability in biomarker levels influenced by factors unrelated to HF, such as renal function, which can affect the specificity and sensitivity of markers like galectin-3 and sST2. Additionally, the cost-effectiveness and standardization of biomarker testing across different healthcare settings remain substantial hurdles to their widespread adoption.

Natriuretic peptides, particularly BNP and NT-proBNP, are currently the cornerstone of HF biomarker utilization, endorsed by guidelines for their diagnostic and prognostic value. However, emerging biomarkers promise to provide a more complete understanding of the disease. These biomarkers offer the potential for a multi-marker strategy, enhancing the accuracy of diagnosis, risk stratification, and monitoring of therapeutic response. Finally, a concerted effort to integrate omics technologies into biomarker research could uncover novel biomarkers and therapeutic targets. These technologies offer the promise of identifying biomarkers that are not only disease-specific but also predictive of therapeutic response, thus paving the way for precision medicine in HF.

## Figures and Tables

**Table 1:** Classification and key features of the main and most-studied heart failure biomarkers.

Category	Examples	Key Features
Natriuretic Peptides (NPs)	B-type natriuretic peptide (BNP), N-terminal pro–B-type natriuretic peptide (NT-proBNP), Atrial natriuretic peptide (ANP)	Reflect cardiac stress; crucial for diagnosing, staging, prognostication, and management of HF
Neurohormonal Activation Biomarkers	Norepinephrine, Chromogranin, Plasma renin activity (PRA), Adrenomedullin (ADM), Mid-regional pro-ADM (MR-proADM)	Indicate severity and predict outcomes in HF; include hormones and enzymes related to the body’s stress response
Cardiac Damage Biomarkers	Cardiac Troponins (TnT and TnI)	Indicate myocardial injury; useful prognostic stratification in acute and chronic HF
Markers of Myocardial Remodeling, Inflammation, and Oxidative Stress	sST2, Galectin-3, GDF 15	Predict mortality and hospitalization risks; reflect structural changes, inflammation, and oxidative stress in the heart

The table provides a detailed overview of the primary heart failure biomarkers, categorizing them based on their pathophysiological origins and highlighting their clinical relevance, offering a comprehensive tool for clinicians in diagnosing and managing heart failure.

**Table 2:** Overview of main heart failure biomarkers, highlighting their advantages, limitations, and current guideline recommendations using ACC/AHA (2017) and ESC (2021).

Biomarker	Advantages	Limitations	Guideline Recommendations
B-type Natriuretic Peptide (BNP) and N-terminal proBNP (NT-proBNP)	Sensitive indicators Reflect hemodynamic changes Prognostic value	Influenced by age, renal function, and obesity Variability in cut-off values	*Diagnosis*: ESC I A ACC/AHA IIa C *Prognosis*: ACC/AHA at admission I A and at discharge IIa B *Screening*: ACC/AHA IIa B
MR-proANP	Stable measure of ANP activity Additive diagnostic value over BNP or NT-proBNP	The role in clinical practice is still evolving	*Diagnosis*: ESC I A
Cardiac Troponins	Indicate myocardial injury High specificity	Elevation can be caused by other cardiac and non-cardiac conditions	*Diagnosis*: ESC I C *Prognosis*: ACC/AHA at admission I A
Galectin-3 Soluble ST2 (sST2)	Reflects fibrosis and inflammation Predictor of remodeling and progression	Elevated in other conditions like renal failure	*Prognosis*: ACC/AHA at admission IIb B

The table examines the biomarkers associated with heart failure, highlighting the advantages, limitations, and variances in their recommendations according to the ACC/AHA and ESC guidelines.

## Data Availability

Not applicable.
